# Low net carbonate accretion characterizes Florida’s coral reef

**DOI:** 10.1038/s41598-022-23394-4

**Published:** 2022-11-15

**Authors:** John T. Morris, Ian C. Enochs, Nicole Besemer, T. Shay Viehman, Sarah H. Groves, Jeremiah Blondeau, Cory Ames, Erica K. Towle, Laura Jay W. Grove, Derek P. Manzello

**Affiliations:** 1 Atlantic Oceanographic and Meteorological Laboratory, Ocean Chemistry and Ecosystem Division, NOAA, 4301 Rickenbacker Cswy., Miami, FL 33149 USA; 2 Cooperative Institute for Marine and Atmospheric Studies, University of Miami, 4600 Rickenbacker Cswy., Miami, FL 33149 USA; 3 National Centers for Coastal Ocean Science, NOAA National Ocean Service, 101 Pivers Island Road, Beaufort, NC 28516 USA; 4 CSS, Inc., Under Contract to NOAA National Centers for Coastal Ocean Science, 10301 Democracy Lane, Suite 300, Fairfax, VA 22030 USA; 5 Southeast Fisheries Science Center, Reef Fish Ecology Unit, NOAA, 75 Virginia Beach Drive, Miami, FL 33149 USA; 6 University of the Virgin Islands, #2 John Brewers Bay, St. Thomas, U.S. Virgin Islands 00802 USA; 7 NOAA Coral Reef Conservation Program, 1305 East-West Highway, Silver Spring, MD 20910 USA; 8 Coral Reef Watch, Center for Satellite Applications and Research, Satellite Oceanography & Climatology Division, U.S. National Oceanic and Atmospheric Administration, College Park, MD 20740 USA

**Keywords:** Ecosystem ecology, Marine biology

## Abstract

Coral reef habitat is created when calcium carbonate production by calcifiers exceeds removal by physical and biological erosion. Carbonate budget surveys provide a means of quantifying the framework-altering actions of diverse assemblages of marine species to determine net carbonate production, a single metric that encapsulates reef habitat persistence. In this study, carbonate budgets were calculated for 723 sites across the Florida Reef Tract (FRT) using benthic cover and parrotfish demographic data from NOAA’s National Coral Reef Monitoring Program, as well as high-resolution LiDAR topobathymetry. Results highlight the erosional state of the majority of the study sites, with a trend towards more vulnerable habitat in the northern FRT, especially in the Southeast Florida region (− 0.51 kg CaCO_3_ m^−2^ year^−1^), which is in close proximity to urban centers. Detailed comparison of reef types reveals that mid-channel reefs in the Florida Keys have the highest net carbonate production (0.84 kg CaCO_3_ m^−2^ year^−1^) and indicates that these reefs may be hold-outs for reef development throughout the region. This study reports that Florida reefs, specifically their physical structure, are in a net erosional state. As these reefs lose structure, the ecosystem services they provide will be diminished, signifying the importance of increased protections and management efforts to offset these trends.

## Introduction

Coral reefs are one of the most biologically diverse ecosystems^1,2^; they support a wealth of ecosystem services (e.g., coastal breakwater protection, commercial fishing, tourism) and are essential sources of income for local economies^3^. The Florida Reef Tract (FRT) is one of the most economically important reef environments in the world, with an estimated value of > $8.5 billion USD^4^. Much of the functionality and ecoservices of coral reefs is dependent on the complex three-dimensional structure of the reef framework^5^.

Reef framework growth and its persistence through time is a function of the balance between calcification (biological precipitation of calcium carbonate [CaCO_3_]) and the breakdown, redistribution and dissolution of CaCO_3_ by physical and biological erosion. Scleractinian corals are often the primary drivers of reef growth and accretion^6^. For healthy reef systems, the balance between these calcifying and bioeroding forces tends to favor net reef accumulation, and with it, positive reef growth^7^.

Despite having limited reef growth over the past 3000 years, the FRT maintained the majority of ecosystem functions up until the late twentieth century due to high regional coral cover^8^. Over recent decades, numerous local and global stressors have contributed to the degradation of the FRT, but disease and bleaching-driven mortality have been the primary driver of losses in live coral^9–12^. Coral cover is low across outer shelf reefs (generally < 5%) in the region, but some reefs closer to shore have maintained higher coral cover and exhibited resilience to recurrent bleaching events^13–16^.

These recent trends have led to two key questions. First, how has the loss in live coral throughout the FRT impacted reef framework growth/loss? Second, what are the implications of the region-wide loss of live coral for net carbonate production in the future?

**Figure 1:**
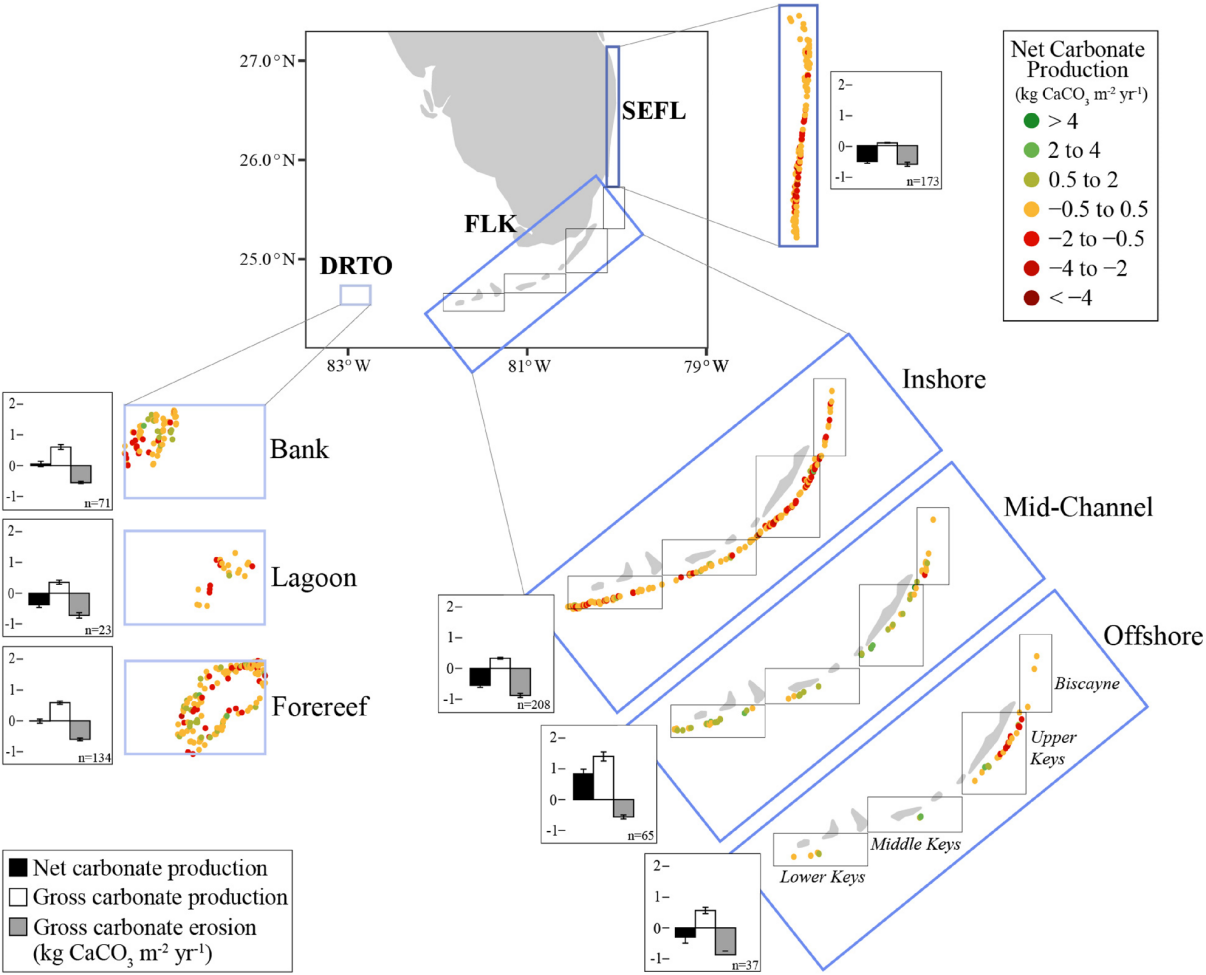
Spatial trends in South Florida reef development. Location of study sites across the FRT with net carbonate production (kg CaCO_3_ m^−2^ year^−1^) represented by the indicated color scheme. The three biogeographic regions (i.e., DRTO, Dry Tortugas; FLK, Florida Keys; and SEFL, Southeast Florida) are separated into individual panels with the reef types specific to that region. No reef type data was available for SEFL. Barplots describe mean net carbonate production (black), gross carbonate production (white), and gross carbonate erosion (grey) for each reef type and sub-region, with n = number of sites. Error bars represent Std. error.

Here we present contemporary rates of reef carbonate accretion and bioerosion measured from 723 coral reef sites across the FRT using a modified *ReefBudget*^17^ approach (Fig. 1). This represents the largest assessment of spatial trends in reef carbonate production across multiple biogeographic regions (n = 3), sub-regions (n = 4) and reef types on the FRT, as well as the largest assessment of reef condition ever conducted for the FRT. This study has major ramifications for the continued delivery of the key ecosystem services provided by coral reefs, such as protection from storms and sea-level rise, as well as commercial and recreational fisheries in the Florida Keys and coastal south Florida.

## Results

Of the three biogeographic regions of the FRT (i.e., Dry Tortugas, Florida Keys, Southeast Florida), the highest mean coral cover was in the Dry Tortugas (DRTO = 7.81% ± 7.83, mean ± SD) and the Florida Keys (FLK = 8.57% ± 10.05) (Table 1). Coral cover in Southeast Florida was very low (SEFL = 1.46% ± 2.27). The FLK had the largest regional variability in coral cover, with cover ranging from 0–52% between sites. DRTO and SEFL had a maximum coral cover of 36% and 12%, respectively.

**Table 1:** A regional/sub-regional comparison of average percent cover of organisms recorded along benthic surveys.

	Region/sub-region					
Percent cover	DRTO	LK	MK	UK	BISC	SEFL
Coral	7.8 (0.5)	10.7 (1.1)	9.6 (1.5)	7.3 (0.8)	5.4 (0.9)	1.5 (0.2)
CCA	2.6 (0.2)	5.1 (0.6)	3.7 (0.7)	4.2 (0.4)	2.3 (0.5)	1.1 (0.2)
Hydrocoral	0.9 (0.1)	0.8 (0.1)	0.8 (0.1)	1.2 (0.1)	0.9 (0.2)	0.3 (0.0)
Macroalgae	41.3 (1.2)	17.4 (1.6)	20.1 (2.6)	27.3 (1.2)	22.2 (3.0)	23.8 (1.4)
Soft Coral	7.3 (0.4)	5.7 (0.4)	3.2 (0.7)	3.6 (0.3)	9.5 (1.7)	8.9 (0.7)
Sponge	6.3 (0.3)	7.4 (0.7)	7.7 (0.7)	4.3 (0.3)	6.9 (1.1)	9.7 (0.6)
Seagrass	0.1 (0.1)	0.2 (0.2)	0.0 (0.0)	1.5 (0.5)	0.8 (0.5)	0.4 (0.2)
Turf Algae	15.6 (1.0)	30.7 (1.6)	30.3 (2.6)	32.2 (1.4)	40.3 (4.2)	38.4 (1.6)
Bare Substrate	14.6 (1.0)	18.6 (1.5)	23.7 (2.9)	17.8 (1.4)	8.8 (2.3)	11.2 (1.3)

Regions/sub-regions are listed in a southern to northern gradient as follows: *DRTO* Dry Tortugas; *LK* Lower Keys; *MK* Middle Keys; *UK* Upper Keys; *BISC* Biscayne; and *SEFL* Southeast Florida. Std. error is listed in parentheses.

### Drivers of net carbonate production/erosion

**Figure 2:**
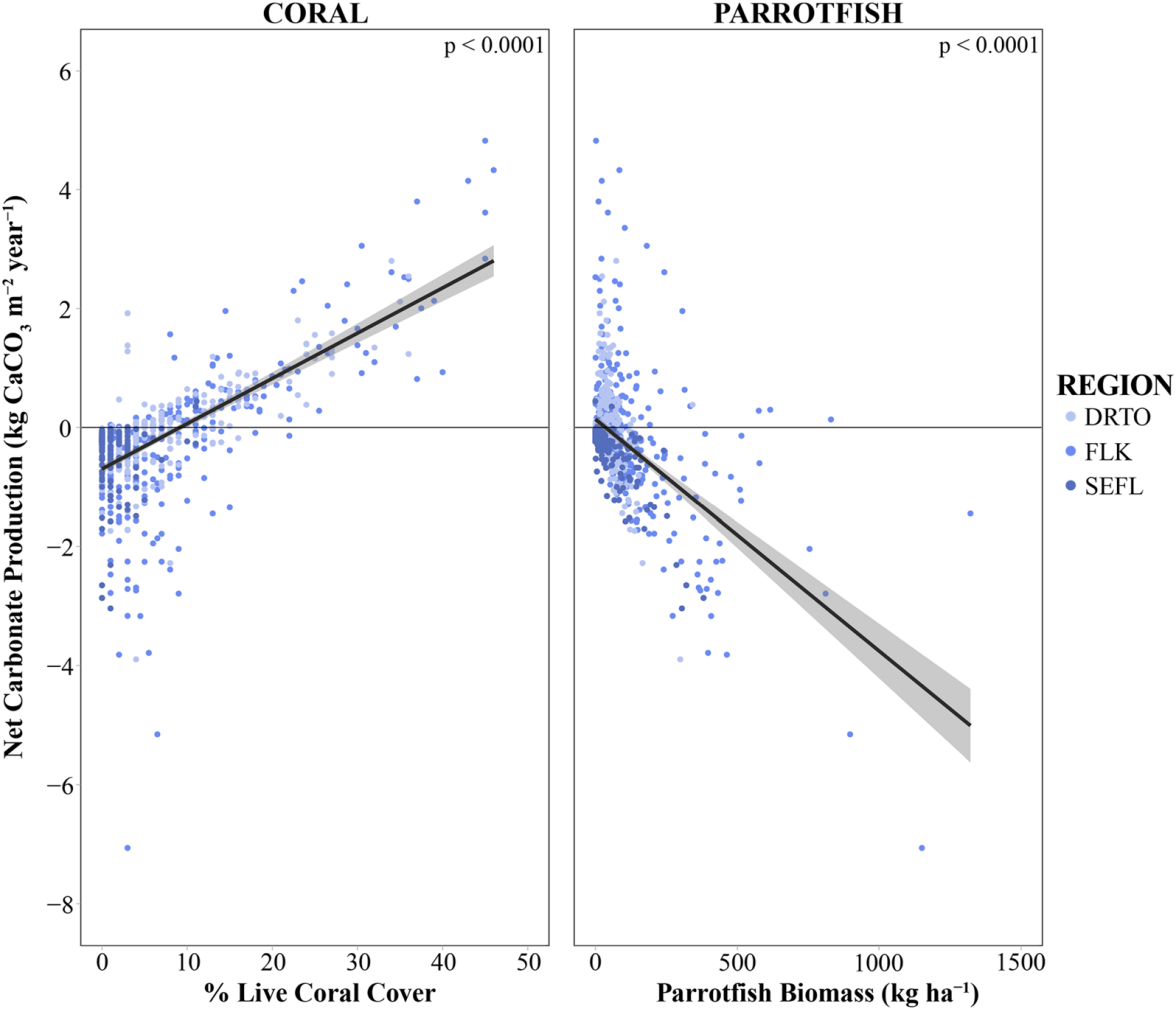
Primary biological drivers of reef development in South Florida. Linear regression plots of net carbonate production (kg CaCO_3_ m^−^^2^ year^−1^) in relation to (**a**) % live coral cover and (**b**) parrotfish biomass (kg ha^−1^) for DRTO, Dry Tortugas (light blue); FLK, Florida Keys (blue); and SEFL, Southeast Florida (dark blue). Data from all three biogeographic regions were pooled together for the statistical analysis. Grey zonation surrounding regression lines represents 95% confidence interval.

There was a significant positive relationship between live coral cover and net carbonate production (linear regression, *R*^2^ = 0.40, *P* < 0.0001, *F* = 479.1) (Fig. 2). The most erosional reefs were found in the FLK and SEFL (minimum net carbonate production of − 7.6 kg CaCO_3_ m^−^^2^ year^−1^ and − 8.5 kg CaCO_3_ m^−^^2^ year^−1^ respectively). Despite having lower average coral cover than the FLK, the minimum recorded net carbonate production in DRTO was − 3.9 kg CaCO_3_ m^−^^2^ year^−1^. Linear regression analysis described a coral cover threshold of 8.2% and 10.5% for DRTO and the FLK, respectively, to maintain positive reef growth (Fig. 2). For SEFL, there was no relationship between coral cover and net carbonate production since reefs in this region were almost exclusively net erosional.

There was a significant negative relationship between parrotfish biomass and net carbonate production (*R*^2^ = 0.28, *P* < 0.0001, *F* = 285.1) (Fig. 2). Parrotfish biomass was significantly different among regions (Generalized linear model [GLM], *F* = 28.3, *P* < 0.0001) and was highest for the FLK reef sites, with an average biomass of 118.7 ± 160.9 kg ha^−1^ (mean ± SD; maximum 1251.3 kg ha^−1^) (Table S1). This is compared to 57.1 ± 93.2 kg ha^−1^ in SEFL (maximum 851.4 kg ha^−1^) and 48.4 ± 40.2 kg ha^−1^ in DRTO (maximum 324.4 kg ha^−1^).

### Regional trends in net carbonate production/erosion

Our results show that reefs in SEFL were almost exclusively net erosional and had the greatest magnitude of erosion of − 0.513 ± 0.063 kg CaCO_3_ m^−^^2^ year^−1^ (mean ± sem) (Fig. 1, Table 2). The Biscayne region (BISC =  − 0.225 ± 0.082 kg CaCO_3_ m^−^^2^ year^−1^) and upper Florida Keys (UK =  − 0.395 ± 0.122 kg CaCO_3_ m^−^^2^ year^−1^) sub-regions were similarly net erosional (Table 2). The highest net carbonate production across the FRT was measured in the MK (0.099 ± 0.095 kg CaCO_3_ m^−^^2^ year^−1^) and LK (0.042 ± 0.128 kg CaCO_3_ m^−^^2^ year^−1^). In comparison to the rest of the FRT, DRTO reefs were found to persist in a state of accretionary stasis, with an average net carbonate production of − 0.023 ± 0.050 kg CaCO_3_ m^−^^2^ year^−1^.

**Table 2:** A regional/sub-regional comparison of average net carbonate production (NCP), gross carbonate production (GCP) and gross carbonate erosion (GCE) of South Florida reef systems in units of kg CaCO_3_ m^-^^2^ year^−1^ (Std. error listed in parentheses).

Region/sub-region	NCP	GCP	GCE
Reef type			
**DRTO**	− 0.023 (0.1)	0.573 (0.0)	− 0.596 (0.0)
Bank	0.057 (0.1)	0.606 (0.1)	− 0.548 (0.0)
Forereef	− 0.007 (0.1)	0.593 (0.1)	− 0.599 (0.0)
Lagoon	− 0.370 (0.1)	0.355 (0.1)	− 0.725 (0.1)
**LK**	0.042 (0.1)	0.808 (0.1)	− 0.767 (0.1)
Inshore	− 0.228 (0.2)	0.640 (0.1)	− 0.868 (0.1)
Mid-Channel	0.895 (0.2)	1.383 (0.2)	− 0.488 (0.1)
Offshore	0.259 (0.2)	0.713 (0.2)	− 0.453 (0.2)
**MK**	0.099 (0.1)	0.573 (0.1)	− 0.474 (0.1)
Inshore	− 0.070 (0.1)	0.388 (0.1)	− 0.458 (0.1)
Mid-Channel	0.642 (0.2)	1.220 (0.2)	− 0.578 (0.2)
Offshore	1.332 (0.8)	1.728 (0.8)	− 0.396 (0.0)
**UK**	− 0.398 (0.1)	0.586 (0.08)	− 0.984 (0.1)
Inshore	− 0.801 (0.1)	0.262 (0.1)	− 1.063 (0.1)
Mid-Channel	1.291 (0.3)	1.941 (0.3)	− 0.649 (0.1)
Offshore	− 0.505 (0.2)	0.507 (0.1)	− 1.014 (0.2)
**BISC**	− 0.225 (0.1)	0.300 (0.1)	− 0.525 (0.1)
Inshore	− 0.374 (0.1)	0.187 (0.0)	− 0.562 (0.1)
Mid-Channel	− 0.006 (0.2)	0.504 (0.1)	− 0.512 (0.1)
Offshore	− 0.201 (0.1)	0.162 (0.1)	− 0.363 (0.2)
**SEFL**	− 0.514 (0.1)	0.092 (0.0)	− 0.606 (0.1)

Sub-regions are listed in a southern to northern gradient as follows: *DRTO* Dry Tortugas; *LK* Lower Keys; *MK* Middle Keys; *UK* Upper Keys; *BISC* Biscayne; and *SEFL* Southeast Florida. A reef type comparison was included for DRTO (i.e., bank, forereef, lagoon) and the FLK region (i.e., inshore, mid-channel, offshore), with the exception of SEFL where reef type classification was absent.

### Shelf position and reef type

Mid-channel reefs throughout the Florida Keys exhibited positive net carbonate production (Table 2). Offshore reefs in the LK and MK, as well as bank reefs in DRTO, also exhibited positive net carbonate production. All other reef types were net erosional (Table 2). Net and gross carbonate production varied significantly between reef types (GLM, *F* = 22.1, *P* < 0.001 and *F* = 29.7, *P* < 0.001, respectively), but gross carbonate erosion did not (Fig. 1, Table 2). In the LK, mid-channel reefs had greater net and gross carbonate production than inshore sites (*F* = 19.6, *P* < 0.0001, and *F* = 21.3, *P* < 0.0001, respectively). The MK showed a clear trend towards increasing net carbonate production further from shore (Fig. 2). Trends related to reef type and reef growth were less clear in BISC, as all habitat types were net erosional (Fig. 1, Table 2).

## Discussion

Of the 723 reef sites analyzed in this study, 70% were net erosional, indicating reef framework is likely being lost across much of the FRT. These results agree with prior reef carbonate budget studies in the Florida Keys that found that 89%^18^ and 77%^11^ of reefs throughout the region were experiencing net erosion. About one-third (37%) of reefs surveyed across several Caribbean/Western Atlantic sites (i.e., Bahamas, Grand Cayman, Bonaire, Belize) were net erosional^19^. This shows that the FRT is in a particularly vulnerable and degraded state when compared to the wider Caribbean.

These data indicate that coral reef habitat throughout the entirety of the FRT is likely losing structural complexity and undergoing a decline in critical ecosystem functions^11,18,20–22^. These reef systems have been negatively altered over the last 50 years, with one of the most consequential changes being a shift away from the dominant habitat-creating coral species (i.e. *Acropora* spp*.* and *Orbicella* spp*.*) to opportunistic “weedy” species (i.e., *Porites astreoides* and *Siderastrea siderea*) with a limited capacity for carbonate production and habitat creation^10,14^. The emergence of these novel, minimal-habitat forming benthic communities are unlikely to reverse the current path of habitat loss and signals that a tipping point towards reef degradation may have already been surpassed for much of the FRT. Associated with these community changes is a greater susceptibility to future environmental perturbations, further deterioration of reef framework^23,24^, and the depreciation of as much as $823 million in annual economic protection value provided by South Florida reef systems^22^.

SEFL, the northernmost biogeographic region in South Florida, is the most degraded region of the FRT. SEFL had the highest percentage of net erosional reefs (98% of reefs), as well as the lowest coral cover and gross carbonate production rates of the FRT (Table 2). SEFL reefs are dominated by non-framework building organisms, such as macroalgae, turf algae, soft corals and sponges, that directly compete with coral for space and are more ephemeral sources of habitat than calcium carbonate reef frameworks^6^. The lack of a sufficient calcifying community has resulted in minimal reef development throughout the region, corroborating past geologic assessments in the area that have estimated a cessation of active upward reef growth 3,700 to 8,000 years ago^8,25,26^.

In comparison to the net erosional state of most SEFL reefs, the mid-channel reefs in the FLK appear to be potential hold-outs for positive net carbonate budgets (Table 2). UK and LK mid-channel reefs may actually offer refugia for future reef development due to elevated gross carbonate production and diminished gross carbonate erosion, indicating that these reefs currently have the most potential to maintain ecoservices. Over the Holocene, the poorest reef development in the FLK was documented for MK reefs^27^, but our data show that the mid-channel and offshore sites in the MK have some of the greatest rates of net carbonate production measured. Future work should investigate if regular exposure to the suboptimal Florida Bay waters in this region has stress-hardened these coral communities and allowed them to persist. It is important to note that the greatest regional gross carbonate production rates documented here (~ 5 kg CaCO_3_ m^−^^2^ year^−1^) are still well below average rates reported from the Caribbean during the Holocene^19^ (≥ 10 kg CaCO_3_ m^−^^2^ year^−1^).

The primary biological drivers of reef development in South Florida were coral cover and parrotfish biomass (Fig. 2). While prior studies have measured a decline in coral cover in the FLK from 12% in 1996 to 4.9% in 2015^24^, this evaluation of South Florida reef systems was marginally more optimistic, with a region-wide average coral cover of 8.2%. However, it is important to note that this value still falls below the estimated 10% coral cover threshold required for sustainable reef development^19^, indicating that South Florida reefs remain in a degraded state.

SEFL reefs had the lowest parrotfish density and second lowest parrotfish biomass measured along the FRT. Associated with this was the smallest percent contribution by parrotfish to gross carbonate erosion, with SEFL being the only region/sub-region to have virtually equal parrotfish and microbioerosion rates (Table S1). These reduced parrotfish populations appear to be uniquely driven by regional benthic cover, with SEFL reef systems largely consisting of low-relief, hard-bottom habitat^28^ marked by only small coral colonies^25,29^. The lack of complex 3D framework not only limits available habitat necessary to sustain robust parrotfish populations, but is also suboptimal substrate for parrotfish feeding due to their preference for convex, rugose substrata^30^.

Specific to the FLK, the parrotfish data supports prior assessments in the region^18^, with the highest parrotfish biomass and erosion rates being measured in the UK and LK. For these sub-regions, inshore and offshore reef types had considerably higher parrotfish biomass compared to the mid-channel systems. This was a main factor driving positive reef development in the mid-channel reefs relative to the net-erosional inshore and offshore counterparts. While previous carbonate budget studies have found significantly higher parrotfish erosion on offshore reef types^31^, the FLK appear to be unique in having substantial inshore and offshore parrotfish populations, with a potential mid-channel respite from parrotfish erosion. Since parrotfish preferentially feed on dead substrate^32^, the low coral cover and high availability of bare substrate on inshore and offshore reefs may promote parrotfish erosion compared to the high coral cover, mid-channel reefs.

This study represents the most comprehensive assessment to date of carbonate production states for the FRT. Net erosion was observed for 70% of sites surveyed; 98% of reefs adjacent to south Florida’s urban centers are eroding. Spatial trends identified the most prominent net erosional reefs in the northernmost part of the FRT, a result that is consistent with prior carbonate budget studies conducted along the Mesoamerican Reef Tract^33^. A comparison of reef types suggest that mid-channel reefs will be potential hold-outs for reef persistence compared to susceptible inshore and offshore reef types, with these trends driven primarily by differences in coral cover and parrotfish biomass. These findings imply that under current conditions, the persistence of FRT habit is in jeopardy unless management strategies intervene to substantially increase carbonate production and coral cover throughout the region.

## Methods

### Survey sites and data collection

Benthic and fish surveys were conducted at randomly stratified sites throughout the entirety of the FRT by NOAA’s National Coral Reef Monitoring Program (NCRMP). Sites were categorized into three biogeographic regions, including Dry Tortugas (DRTO, n = 228), Florida Keys (FLKs, n = 322), and Southeast Florida (SEFL, n = 173) (Fig. 1). The Florida Keys were further classified into the following four sub-regions: Lower Keys (LK, n = 103), Middle Keys (MK, n = 46), Upper Keys (UK, n = 140), Biscayne (BISC, n = 33). Within each region/sub-region (except for SEFL), reefs were categorized according to reef types. For DRTO, this included bank, forereef, and lagoon reef sites. For the LK, MK, UK, and BISC, reef types were categorized as inshore, mid-channel, and offshore. Data were collected throughout the region in 2014, 2016, and 2018.

Fish and benthic surveys were conducted in accordance with NCRMP methodologies^34^ (Table S2). The protocol used for the fish surveys was developed from a modified Reef Visual Census (RVC) method^35^ and was performed using a stratified random sampling design. Divers surveyed two 15 m diameter cylinders, spaced 15 m apart. Fish species were identified to the lowest taxonomic level for a period of five minutes. This was followed by an additional five minutes dedicated to recording species abundances and sizes (10 cm bins).

Surveys were used to quantify the benthic cover at each site. The protocol for these surveys followed a standard line point-intercept sampling design. At each site, a 15 m weighted transect was draped along the reef surface. Surveyors recorded benthic composition at 15 cm intervals along the transect (i.e., 100 equidistant points). The benthic composition from these 100 points was then transformed to percent cover of ecologically important functional groups (scleractinian coral [species-specific], gorgonians, hydrocoral, CCA, macroalgae, turf algae, sponges, bare/dead substrate, sand/sediment).

### Carbonate budget analysis

Planar benthic surveys were adjusted to account for the three-dimensional complexity (i.e., rugosity) of each site using light detection and ranging (LiDAR) data (1 m horizontal resolution; 15 cm vertical resolution) from topobathymetric mapping surveys of the South Florida eastern coastline conducted by NOAA’s National Geodetic Survey. A 15 m x 15 m region of interest (ROI) was placed around the GPS coordinates of each site using ArcGIS Pro with 3D and Spatial Analyst extensions (ESRI). The ROI was then overlaid with existing multibeam echosounder (MBES) and LiDAR bathymetry data. Within the ROI, LiDAR was extracted using the Clip Raster function from ArcPy (ArcGIS’s python coding interface), and the Surface Volume tool was used to calculate the 3D surface area. Rugosity was calculated by dividing the 3D surface area by the 2D surface area of the ROI.

The methodology for standardizing reef carbonate budgets to topographic complexity (i.e., rugosity) diverged from that of the *ReefBudget* approach by using site-specific rugosity rather than species-specific rugosity^17^. This was a necessary limitation of this analysis as transect rugosity at 1 m increments was not measured using the NCRMP benthic survey protocol. To ensure that reef topographic complexity was still accounted for, however, rugosity of the entire reef site, calculated from LiDAR bathymetry data, was used in this analysis. While rugosity of the site rather than of each benthic component, specifically for corals, can lead to an under or overestimation of carbonate production rates, we note that site and species rugosity (i.e., encrusting and massive coral morphologies) was low for the vast majority of sites and species surveyed, thereby reducing the probability of an under or overestimation.

Reef carbonate budget analysis was performed following a modified version of the *ReefBudget* approach^17^. Coral carbonate production was derived from species-specific linear extension rates (cm year^−1^), skeletal density (g cm^−3^), coral morphology (branching, massive, sub-massive, encrusting/plating), and percent cover. Carbonate production by CCA and other calcareous encrusters was similarly calculated as a function of surface area, literature reported linear extension rates, and skeletal density^17^. Gross carbonate production at each survey site was measured as the sum total of carbonate production by all calcareous organisms found at each site and was standardized to site-specific reef rugosity.

Gross carbonate erosion for each survey-site was calculated as the sum total of erosion by four bioeroding groups: parrotfish, microborers, macroborers, and urchins. The calculations roughly followed the *ReefBudget* methodologies^17^ (Table 1). Parrotfish size frequency distributions from NCRMP surveys were multiplied by size and species-specific bite rates (bites min^−1^), volume removed per bite (cm^3^), and proportion of bites leaving scars to calculate total parrotfish erosion^17^. The substrate density (1.72 g cm^−3^) used in these calculations followed that of the *ReefBudget* protocol^17^. Microbioerosion was calculated from the percent cover of dead coral substrate, which was multiplied by a literature-derived rate^17^ of − 0.240 kg CaCO_3_ m^−^^2^ year^−1^. Macroboring was calculated as the percent cover of clionid sponges multiplied by the average erosion rate of all Caribbean/Atlantic clionid sponges^17^ (-6.05 kg CaCO_3_ m^−^^2^ year^−1^). External bioerosion by urchins was calculated using *Diadema* urchin abundance collected from the benthic surveys. Due to the lack of test size data from the NCRMP benthic surveys, urchin abundance was multiplied by the bioerosion rate of an average test sized^36^ (66 mm) Caribbean/Atlantic *Diadema* urchin (-0.003 kg CaCO_3_ m^-^^2^ year^−1^). While using an average test sized *Diadema* urchin for this analysis may have led to an under or overestimation of urchin erosion, the abundance of *Diadema* urchins measured in the surveys was minimal, as they appeared to be functionally irrelevant across the FRT.

### Model validation

As the survey methodologies and data sources employed in this analysis were modified from that of the standard *ReefBudget* approach^17^, we chose to validate our model through a fine scale temporal comparison of annual *ReefBudget* surveys conducted by NOAA at Cheeca Rocks (UK) to three nearby NCRMP sites used in our analysis. Since the NCRMP surveys were performed in 2014, 2016, and 2018, this study focused exclusively on these three survey years from the NOAA Cheeca Rocks dataset. Temporal trends related to reef growth/erosion were visually compared to see if survey types provided comparable results (SI Figure S6).

### Statistical analysis

All model calculations and statistical analyses were performed using R^37^ with the R Studio extension^38^. Generalized linear models (GLMs) were run on response variables involved in habitat production (i.e., net carbonate production, gross carbonate production, and gross carbonate erosion) to evaluate spatial trends related to reef development across sub-regions and reef types. Each GLM was performed with reef type being nested within sub-region. The best fit distribution for each variable was determined using the fitdistrplus R package^39^. Linear regression analysis was used to evaluate the relationship between net carbonate production and both live coral cover and parrotfish biomass. All plots were created using ggplot2 R package^40^ and edited for style with Adobe Illustrator^41^.

## Supplementary Information

Supplementary Information.

## Data Availability

The datasets generated during and/or used for the analysis of the current study are publicly available at NCEI and ERDDAP: https://coastalscience.noaa.gov/project/national-coral-reef-monitoring-program-biological-socioeconomic/.
