# Supplementary material for: Low net carbonate accretion characterizes Florida’s coral reef
Source: Sci Rep. 2022 Nov 15;12:19582. doi: 10.1038/s41598-022-23394-4 (PMC9666464; doi:10.1038/s41598-022-23394-4)
Supplement: Supplementary file 1 — Supplementary Information. [file 41598_2022_23394_MOESM1_ESM.docx]

**TITLE:** Low Net Carbonate Accretion Characterizes Florida’s Coral Reef

**AUTHOR LIST:** Morris, John T.^a,b*^; Enochs, Ian C.^a^; Besemer, Nicole^a,b^; Viehman, T. Shay^c^; Groves, Sarah H.^d^; Blondeau, Jeremiah^e,f^; Ames, Cory^d^; Towle, Erica K.^g^; Grove, Laura Jay W.^e^; Manzello, Derek P.^h^

^a^NOAA, Atlantic Oceanographic and Meteorological Laboratory, Ocean Chemistry and Ecosystem Division, 4301 Rickenbacker Cswy., Miami, FL 33149, USA

^b^University of Miami, Cooperative Institute for Marine and Atmospheric Studies, 4600 Rickenbacker Cswy., Miami, FL 33149, USA

^c^NOAA National Ocean Service, National Centers for Coastal Ocean Science, 101 Pivers Island Road, Beaufort, NC 28516, USA

^d^CSS, Inc., under contract to NOAA National Centers for Coastal Ocean Science, 10301 Democracy Lane, Suite 300, Fairfax, Virginia 22030, USA

^e^NOAA, Southeast Fisheries Science Center, Reef Fish Ecology Unit, 75 Virginia Beach Drive, Miami, FL 33149, USA

^f^University of the Virgin Islands, #2 John Brewers Bay, St. Thomas, U.S. Virgin Islands 00802, USA

^g^NOAA Coral Reef Conservation Program, 1305 East-West Highway, Silver Spring, MD 20910, USA

^h^Coral Reef Watch, Center for Satellite Applications and Research, Satellite Oceanography & Climatology Division, U.S. National Oceanic and Atmospheric Administration, College Park, MD 20740, USA

**Supplemental Material**

**Table S1** | A regional/sub-regional comparison of average net carbonate production (NCP), gross carbonate production (GCP) and gross carbonate erosion (GCE) of South Florida reef systems in units of kg CaCO_3_ m^-2^ year^-1^. Regions/sub-regions are listed in a southern to northern gradient as follows: DRTO, Dry Tortugas; LK, Lower Keys; MK, Middle Keys; UK, Upper Keys; BISC, Biscayne; and SEFL, Southeast Florida. Individual contributions by calcifiers (coral, CCA, hydrocoral) and bioeroders (parrotfish, macrobioerosion, microbioerosion, diadema) to GCP and GCE are also compared between regions and sub-regions.

|  | **Region/Sub-Region** | | | | | |
| --- | --- | --- | --- | --- | --- | --- |
| **Parameter** | **DRTO** | **LK** | **MK** | **UK** | **BISC** | **SEFL** |
| NCP | -0.023 | 0.042 | 0.099 | -0.398 | -0.225 | -0.514 |
| GCP | 0.573 | 0.809 | 0.573 | 0.586 | 0.300 | 0.092 |
| Coral | 0.557 | 0.787 | 0.555 | 0.563 | 0.285 | 0.086 |
| CCA | 0.006 | 0.013 | 0.009 | 0.010 | 0.006 | 0.003 |
| Hydrocoral | 0.010 | 0.009 | 0.009 | 0.013 | 0.010 | 0.003 |
| GCE | -0.596 | -0.767 | -0.474 | -0.984 | -0.530 | -0.606 |
| Parrotfish | -0.377 | -0.551 | -0.265 | -0.771 | -0.329 | -0.319 |
| Macrobioerosion | <0.000 | <0.000 | <0.000 | <0.000 | <0.000 | <0.000 |
| Microbioerosion | -0.217 | -0.215 | -0.208 | -0.213 | -0.194 | -0.286 |
| Diadema | -0.003 | -0.001 | -0.001 | -0.001 | -0.001 | 0.000 |

**Table S2 |** Model parameters utilized in the carbonate budget analysis of South Florida reef systems, along with the geographic region where the parameters were collected (i.e., Region) and the source of the data (i.e., Reference).

| **Parameter** | **Region** | **Reference** |
| --- | --- | --- |
| Coral |  |  |
| % Cover | Florida Keys | NCRMP |
| Calcification | Atlantic/Caribbean | Perry et al. 2012 |
| CCA |  |  |
| % Cover | Florida Keys | NCRMP |
| Calcification | Generalized | Perry et al. 2012 |
| Endolithic Algae |  |  |
| % Cover | Florida Keys | NCRMP |
| Bioerosion | Atlantic/Caribbean | Perry et al. 2012 |
| Clionid sponges |  |  |
| % Cover | Florida Keys | NCRMP |
| Bioerosion | Atlantic/Caribbean | de Bakker et al. 2018 |
| Parrotfish |  |  |
| Species, size, abundance | Florida Keys | NCRMP |
| Bioerosion | Atlantic/Caribbean | Perry et al. 2012 |
| *Diadema* |  |  |
| Abundance | Florida Keys | NCRMP |
| Test size | Florida Keys | Feehan et al. 2016 |
| Bioerosion | Atlantic/Caribbean | Perry et al. 2012 |

**Table S3** **|** Average coral cover (%) and carbonate production (kg CaCO_3_ m^-2^ year^-1^) for the five most abundant coral species within each region/sub-region. Regions/sub-regions are listed in a northern to southern gradient as follows: SEFL, Southeast Florida; BISC, Biscayne; UK, Upper Keys; MK, Middle Keys; LK, Lower Keys; and DRTO, Dry Tortugas. Std. dev is listed in parentheses.

| **Region/Sub-Region** | **% Coral Cover** | **Carbonate Production** |
| --- | --- | --- |
| Species |  | (kg CaCO_3_ m^-2^ year^-1^) |
| **SEFL** |  |  |
| *Orbicella franksi* | 3.25 (1.5) | 0.22 (0.1) |
| *Siderastrea siderea* | 2.19 (0.4) | 0.12 (0.0) |
| *Montastraea cavernosa* | 2.11 (0.3) | 0.13 (0.0) |
| *Porites porites* | 1.66 (0.7) | 0.08 (0.0) |
| *Porites astreoides* | 1.38 (0.2) | 0.09 (0.0) |
| **BISC** |  |  |
| *Acropora cervicornis* | 3.37 (0.9) | 2.45 (0.6) |
| *Orbicella faveolata* | 2.66 (0.6) | 0.32 (0.1) |
| *Siderastrea siderea* | 2.54 (0.3) | 0.14 (0.0) |
| *Porites porites* | 2.41 (0.5) | 0.12 (0.0) |
| *Dichocoenia stokesii* | 2.13 (0.7) | 0.05 (0.0) |
| **UK** |  |  |
| *Orbicella faveolata* | 6.41 (1.7) | 0.76 (0.2) |
| *Colpophyllia natans* | 4.75 (2.8) | 0.24 (0.1) |
| *Siderastrea siderea* | 3.65 (0.4) | 0.21 (0.0) |
| *Orbicella annularis* | 2.75 (0.5) | 0.43 (0.1) |
| *Pseudodiploria strigosa* | 2.42 (0.4) | 0.15 (0.0) |
| **MK** |  |  |
| *Siderastrea siderea* | 4.25 (0.5) | 0.24 (0.0) |
| *Colpophyllia natans* | 3.93 (0.8) | 0.20 (0.0) |
| *Dichocoenia stokesii* | 2.83 (1.7) | 0.07 (0.0) |
| *Montastraea cavernosa* | 2.83 (0.5) | 0.18 (0.0) |
| *Orbicella faveolata* | 2.31 (0.4) | 0.28 (0.0) |
| **LK** |  |  |
| *Orbicella annularis* | 7.23 (2.9) | 1.10 (0.4) |
| *Orbicella faveolata* | 4.85 (0.7) | 0.58 (0.1) |
| *Colpophyllia natans* | 4.33 (0.6) | 0.22 (0.0) |
| *Siderastrea siderea* | 4.11 (0.3) | 0.23 (0.0) |
| *Montastraea cavernosa* | 3.54 (0.5) | 0.22 (0.0) |
| **DRTO** |  |  |
| *Orbicella franksi* | 3.93 (0.5) | 0.27 (0.0) |
| *Orbicella faveolata* | 3.72 (0.5) | 0.45 (0.1) |
| *Montastraea cavernosa* | 3.51 (0.3) | 0.22 (0.0) |
| *Colpophyllia natans* | 3.11 (0.4) | 0.16 (0.0) |
| *Agaricia lamarcki* | 2.86 (0.6) | 0.03 (0.0) |

**Table S4 |** Average parrotfish biomass (kg ha^-1^) and bioerosion (kg CaCO_3_ m^-2^ year^-1^) for the four most abundant parrotfish species within each region/sub-region. Regions/sub-regions are listed in a northern to southern gradient as follows: SEFL, Southeast Florida; BISC, Biscayne; UK, Upper Keys; MK, Middle Keys; LK, Lower Keys; and DRTO, Dry Tortugas. Std. dev is listed in parentheses.

| **Region/Sub-Region** | **Biomass** | **Bioerosion** |
| --- | --- | --- |
| Species | (kg ha^-1^) | (kg CaCO_3_ m^-2^ year^-1^) |
| **SEFL** |  |  |
| *Sparisoma aurofrenatum* | 7.89 (13.69) | 0.002 (0.00) |
| *Sparisoma viride* | 10.73 (27.99) | 0.176 (0.44) |
| *Scarus iseri* | 3.15 (9.69) | 0.005 (0.02) |
| *Scarus taeniopterus* | 4.54 (10.08) | 0.014 (0.03) |
| **BISC** |  |  |
| *Sparisoma aurofrenatum* | 7.70 (12.17) | 0.002 (0.00) |
| *Sparisoma viride* | 15.08 (25.98) | 0.254 (0.43) |
| *Scarus iseri* | 4.17 (6.34) | 0.005 (0.01) |
| *Scarus taeniopterus* | 2.23 (5.53) | 0.007 (0.02) |
| **UK** |  |  |
| *Sparisoma aurofrenatum* | 8.30 (11.38) | 0.002 (0.00) |
| *Sparisoma viride* | 17.48 (28.44) | 0.294 (0.47) |
| *Scarus iseri* | 3.67 (5.55) | 0.004 (0.01) |
| *Scarus taeniopterus* | 3.12 (6.62) | 0.010 (0.02) |
| **MK** |  |  |
| *Sparisoma aurofrenatum* | 5.53 (7.10) | 0.002 (0.00) |
| *Sparisoma viride* | 10.51 (26.47) | 0.174 (0.43) |
| *Scarus iseri* | 3.72 (5.42) | 0.005 (0.01) |
| *Scarus taeniopterus* | 3.64 (8.90) | 0.011 (0.03) |
| **LK** |  |  |
| *Sparisoma aurofrenatum* | 5.89 (9.36) | 0.002 (0.00) |
| *Sparisoma viride* | 15.65 (33.92) | 0.252 (0.52) |
| *Scarus iseri* | 4.00 (5.27) | 0.005 (0.01) |
| *Scarus taeniopterus* | 2.13 (6.47) | 0.006 (0.02) |
| **DRTO** |  |  |
| *Sparisoma aurofrenatum* | 5.29 (7.01) | 0.001 (0.00) |
| *Sparisoma viride* | 11.69 (19.26) | 0.197 (0.32) |
| *Scarus iseri* | 3.94 (3.93) | 0.004 (0.01) |
| *Scarus taeniopterus* | 0.69 (2.52) | 0.002 (0.01) |

**
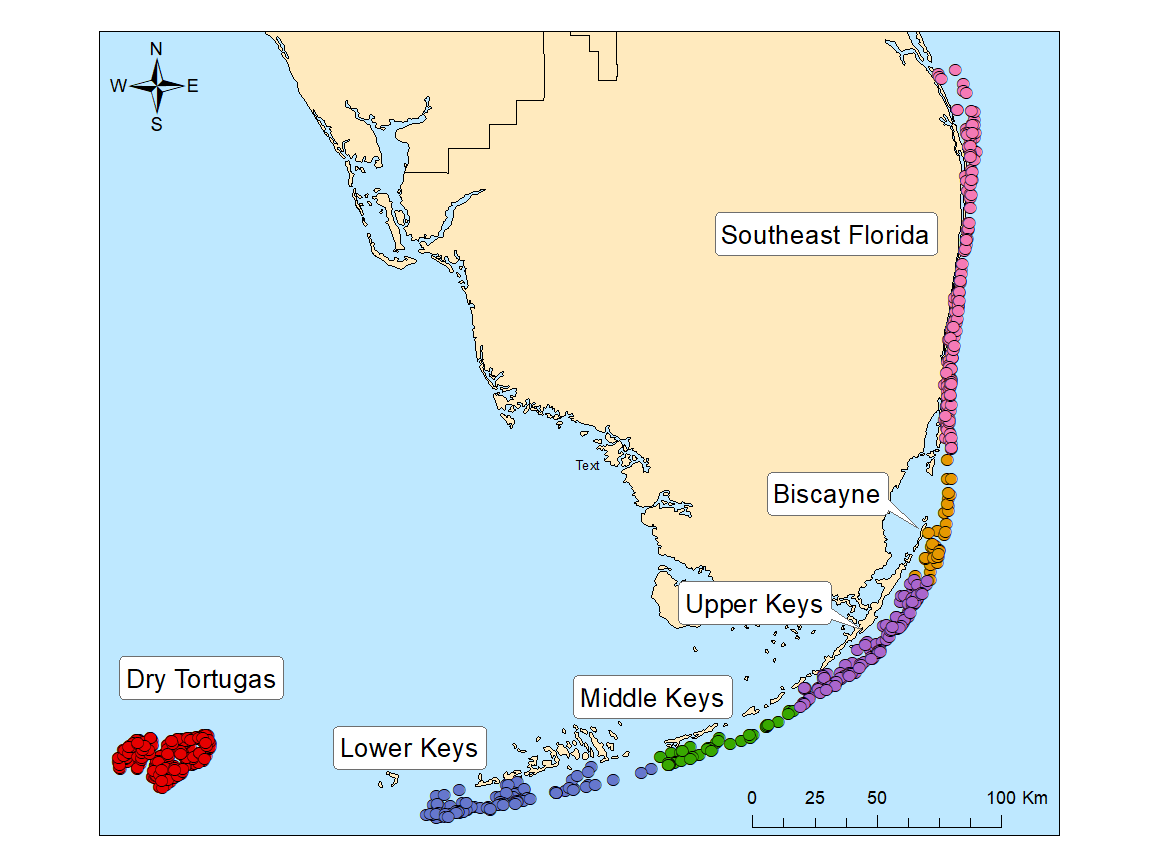
**

**Figure S1 |** Map showing the distribution of reef sites analyzed during this study. Sites were categorized in sub-regions along a south to north gradient: Dry Tortugas (DRTO, n = 228), Lower Keys (LK, n = 103), Middle Keys (MK, n = 46), Upper Keys (UK, n = 140), Biscayne (BISC, n = 33), and Southeast Florida (SEFL, n = 173). For regional comparisons, LK, MK, UK, and BISC were grouped into the FLK and compared to DRTO and SEFL.

**Figure S2 |** Maps showing the distribution of the LiDAR surveys (grey) and location of each reef site (green) along the FRT (a, b). A 15 m x 15 m grid was wrapped around each reef site’s GPS point (c), and rugosity was calculated as 3D surface area/2D surface area.

**Figure S3** | A regional/sub-regional comparison of net carbonate production (kg CaCO_3_ m^-2^ year^-1^; black), gross carbonate production (white) and gross carbonate erosion (grey) for DRTO, Dry Tortugas; LK, Lower Keys; MK, Middle Keys; UK, Upper Keys; BISC, Biscayne; SEFL, Southeast Florida. Error bars represent Std. error.


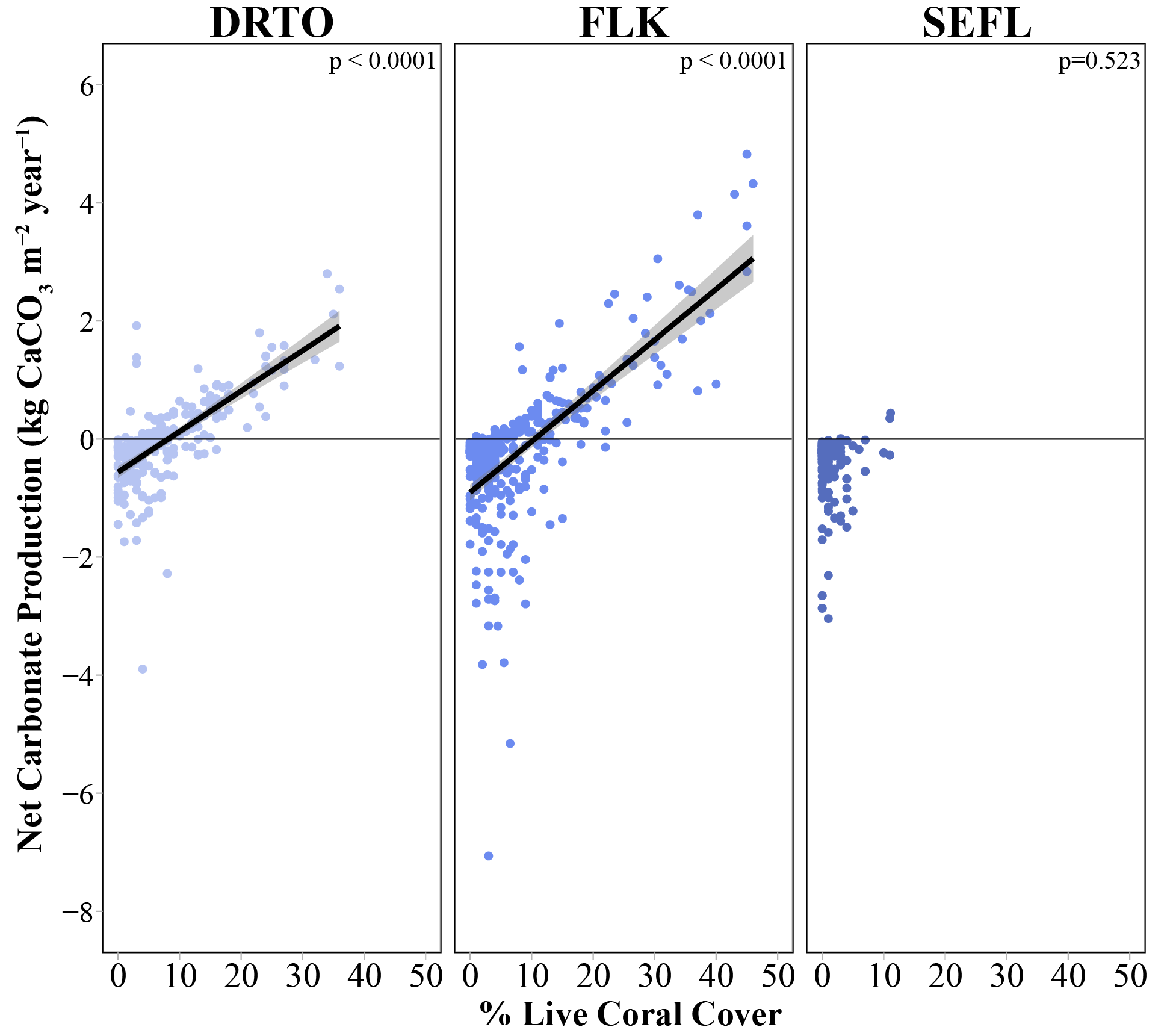


**Figure S4** | The relationship between regional % live coral cover for each site as a function of net carbonate production (kg CaCO_3_ m^-2^ year^-1^). The point at which the DRTO (Dry Tortugas) and FLK (Florida Keys) linear regression lines pass the x-axis represents the minimum % live coral cover required to maintain positive reef development (8.2% for DRTO, 10.5% for FLK). Grey zonation surrounding the regression lines represents 95% confidence interval.

**Figure S5** | The relationship between regional/sub-regional % live coral cover for each site as a function of net carbonate production (kg CaCO_3_ m^-2^ year^-1^). Regions/sub-regions are listed in a southern to northern gradient (top-left to bottom-right) as follows: DRTO, Dry Tortugas; LK, Lower Keys; MK, Middle Keys; UK, Upper Keys; BISC, Biscayne; and SEFL, Southeast Florida. Red zonation surrounding the regression lines represents 95% confidence interval.

**
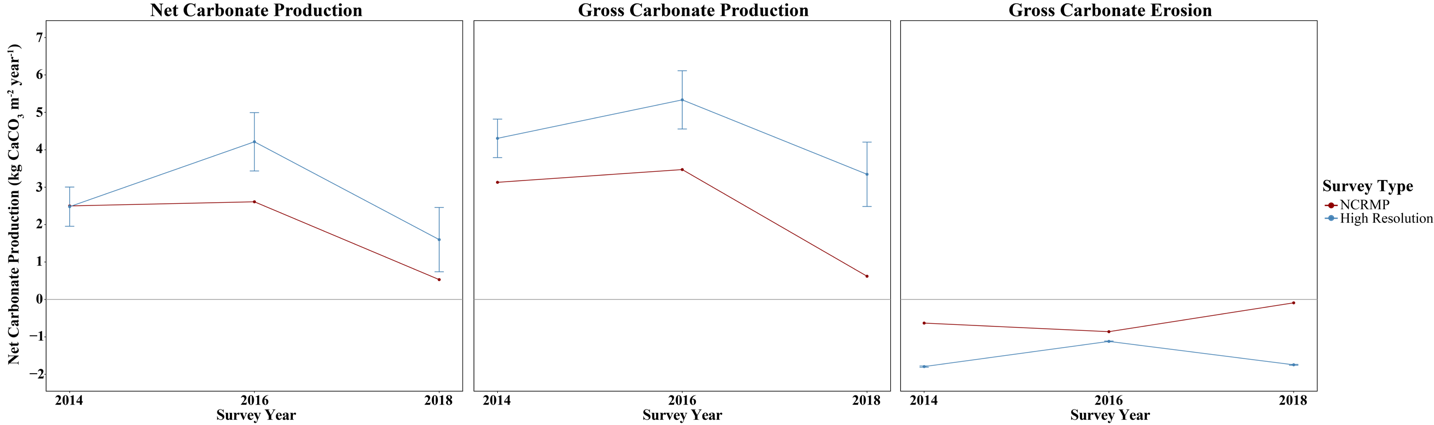
**

**Figure S6 |** Fine-scale comparison of net carbonate production (kg CaCO_3_ m^-2^ year^-1^), gross carbonate production (kg CaCO_3_ m^-2^ year^-1^), and gross carbonate erosion (kg CaCO_3_ m^-2^ year^-1^) at Cheeca Rocks (UK) reef sites as a function of survey year (2014, 2016, 2018). Comparison of NCRMP (red line) and high-resolution NOAA (blue line) *ReefBudget* surveys can be visualized within each panel and were used to validate the NCRMP datasets as it relates to differences in survey design from the *ReefBudget* approach. High-resolution NOAA *ReefBudget* surveys were conducted across six transects, with net carbonate production calculated as the average of the six transects. NCRMP surveys were conducted across one transect for each survey year. Error bars for NOAA *ReefBudget* surveys represent Std. deviation.
